# Evaluating the long-term care insurance policy from medical expenses and health security equity perspective: evidence from China

**DOI:** 10.1186/s13690-021-00761-7

**Published:** 2022-01-04

**Authors:** Huan Liu, Tiantian Hu

**Affiliations:** 1 Zhejiang University of Finance & Economics, Hang Zhou, China; 2 Wuhan University, Wuhan, China

**Keywords:** Long-term care insurance, Disability, Medical expenses, Health level, DID

## Abstract

**Background:**

Since the national long-term care (LTCI) policy pilot in 2016 of China, the LTCI policy has had significant impact on the residents in the pilot area.

**Methods:**

From the perspective of medical expenses and health security equity, this study selects tracking survey data from the CHARLS database in 2013, 2015, and 2018 and empirically investigates the effect of LTCI policy pilot by using differences-in-differences method (DID). Moreover, this study measures the economic distribution and health equity of the treated and untreated groups using the concentration and Theil indices.

**Results:**

The results showed that group heterogeneity of medical expenses and health level of elderly in the treatment group were narrowing. Moreover, the policy results showed that the LTCI policy pilot significantly affects the outpatient, hospital expenses, and length of stay of elders. Residence registration, income level, and basic medical insurance play a significant regulatory role. Additionally, LTCI policy pilot significantly improved the overall health of the elderly.

**Conclusions:**

The measurement results of inequality show that the policy increases the income of low-income people, lowers the inequality level of outpatient and inpatient reimbursement, and reduces the concentration index of ADL disability and serious diseases. However, the inequality of serious diseases is becoming higher. Based on this, this paper provides several suggestions on optimizing the pilot policy of LTCI.

## Background

According to the data of China’s Seventh National Population Census, by the end of 2020, China’s population that aged 60 and over had reached 264 million, accounting for 18.7% of the total population. Among them, the population aged 65 and over is 191 million, accounting for 13.50% of the total population [1]. China has entered into an aging society. With the growth of age, the burden borne by the current family and public medical systems will continue increasing. The aging trend will continue to bring huge medical and socio-economic challenges to decision makers and caregivers, and the main users of the public medical system [2]. For example, the one-child policy, the migration of rural population to cities and the expansion of the population of “empty nest” elderly have had significant impact on traditional family care, further aggravating the burden of current public health care [3]. From the existing research, the intensification of population aging has a direct impact on public health care. On the one hand, allocating the medical resources more effectively, and establishing a security policy for the life care of the elderly population are necessary. On the other hand, improving the sharing mechanism to inhibit the growth of public medical burden is vital.

In this context, some developed countries have also begun piloting long-term care policy pilot (LTCI) systems, such as Germany and Japan. LTCI refers to the core orientation of social insurance. These systems primarily allow individuals who are disabled because of old age, disease, or disability to obtain care from the state or society, or medical care cost compensation, or service guarantee closely related to basic life. LTCI mainly includes basic life care services and medical rehabilitation care services [4, 5]. Among them, basic life care services include assistance in eating, bathing, changing clothes, urination management, safety care, and so on. Medical rehabilitation care services include muscle exercise, pressure ulcer prevention and care, and so on. To actively face the growing risks of aging and disability, China began piloting LTCI policies in 15 regions in 2016. However, great commonalities in the LTCI pilot process exist. First, overall, all pilot areas have not achieved full coverage of urban and rural residents. Second, regional entities rely on basic medical insurance for financing; hence, participation is in the form of direct coverage. Third, on treatment level, national standards require the overall reimbursement rate to be maintained at 70%, while specific projects to be provided according to local standards and a specific ceiling line [6, 7]. Additionally, the current policy pilot still faces practical difficulties such as the unbalanced development of nursing service institutions, community and family nursing services, the shortage and low quality of nursing staff, and fragmented financing channels of LTCI [8]. Since the financing channels in LTCI pilot cities rely on the basic health care insurance system, combined with the data of the first batch of 15 LTCI pilot cities in China, an empirical analysis of the LTCI pilot effect from the perspective of residents’ medical consumption and health level is an effective evaluation of the existing LTCI pilot policies. Moreover, it is the key to exploring the coordinated development of health care before and after implementation of LTCI. The changes of policy framework are shown in Table 1.

**Table 1:**
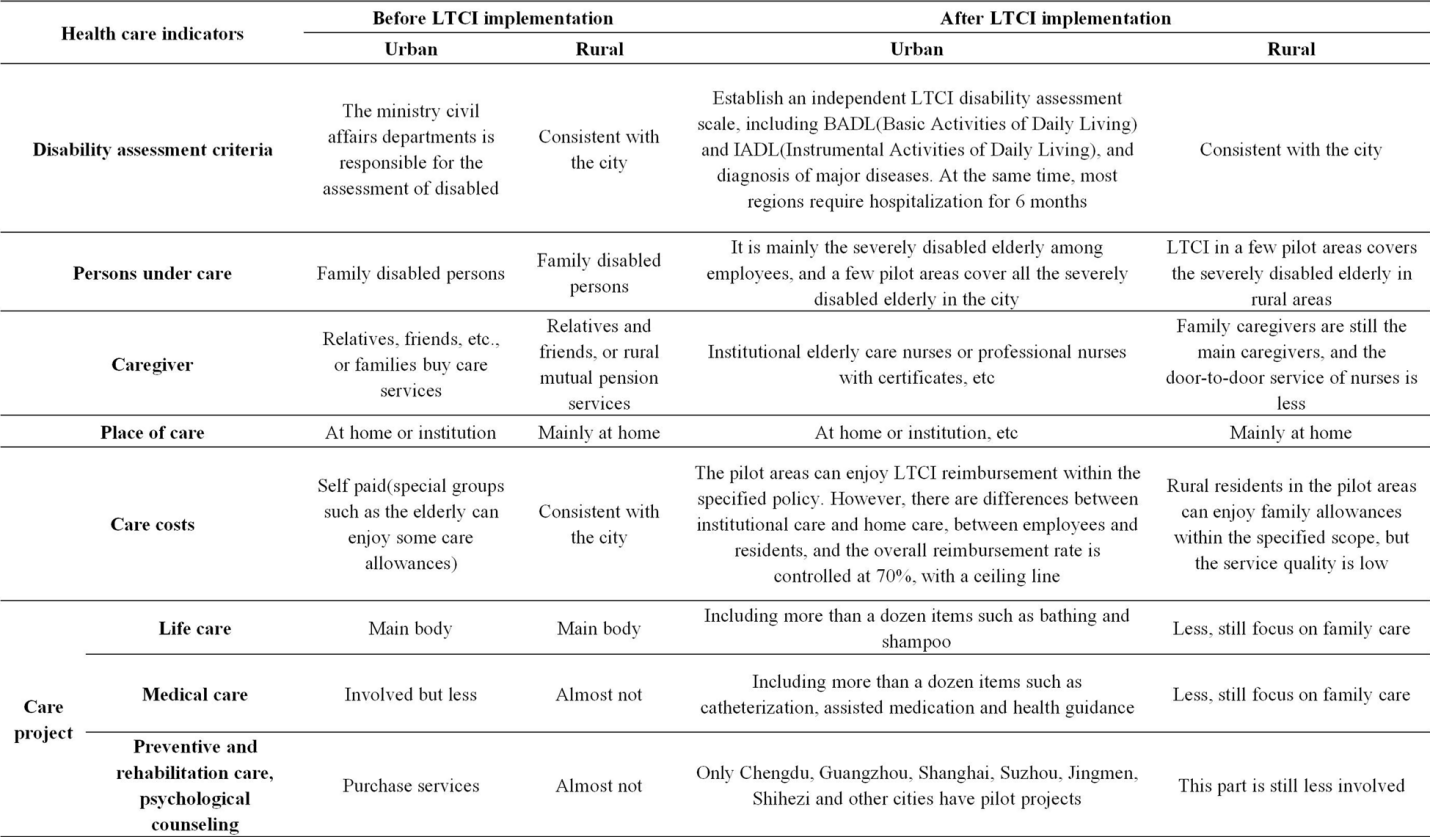
Comparison of heath care before and after the implementation in the 15 LTCI first pilot cities in China

## Literature review

While social policy is intended for solving social problems, it is also a social problem. Considering the LTCI as an important tool of the formal system, it can be found that there are many discussions on the relationship between formal and informal care for the disabled elderly in China [9–12]. On the existing research on the pilot effect of the LTCI policy, foreign research remains relatively rich and comprehensive.

Additionally, from existing research on the pilot effect of LTCI policy, research conclusions mostly focus on both economic and health effects. First, on economic effects, while existing studies have mainly focused on the control of medical expenses [13–16]. Moreover, the existing research conclusions can be divided into two categories, namely, alternative medical expenses and release medical expenses [17]. First, the economic effect of LTCI is mainly for replacing medical expenses. For example, Choi et al. [18] found that compared with non-beneficiaries of LTCI, the number of inpatients of beneficiaries is significantly reduced, and their length of stay is also significantly shortened. Ma et al. [13] found that LTCI effectively saves the expenditure of the medical insurance fund. The second effect is the release of medical expenses. Research shows that in developed countries, no matter what model of LTCI policy is adopted, the total expenses of LTCI in various countries is rising [6, 19–23]. In terms of personal burden, most countries that implementing LTCI need beneficiaries to pay a certain proportion of their own expenses [24–26], and the high proportion of individual out-of-pocket expenses or long-term care (LTC) expenses have an important impact on the personal burden of beneficiaries [7, 27, 28].

Comparatively, few studies concerning health effects exist, and scholars have not reached a consensus on the health effects of LTCI. On the one hand, positive health effects of LTCI are proved. For example, Yasutake et al. [29] studied the effectiveness of LTCI intervention policy from the perspective of OHN (Oral Health and Nutrition) program in Japan’s LTCI system. Their results show that the OHN plan is expected to reduce accidental disability and reduce medical expenses. Guided by the concept of “Value-Based Health Care,” Ma et al. [13] found that the LTCI policy improved the mental health level of disability objects and reduced physical pain to a certain extent. On the other hand, negative health effects of LTCI are observed. Medical security or nursing service policies are not as important as expected [30, 31], and, compared with factors such as gene, environment, region and income, the health effect of medical security is actually quite small [32, 33]. From the health behavior perspective, after implementation of LTCI, whether the elderly can improve their health through the utilization of LTC services has been studied [5, 34]. Moreover, Nemoto et al. [35] considered the difference in physical vulnerability of people covered by LTCI as the object and found that the physical function of vulnerable elderly people became worse than before.

Because of the differences in research methods and data, such as differences-in-differences (DID) method, propensity matching and quantile regression methods, many inconsistencies in the research conclusions of the effect of the existing LTCI policy can be found. In addition, on the effect of China’s LTCI policy, scholars have mainly focused on the economic effect. Moreover, as the policy beneficiary groups in the pilot areas are mainly severely disabled individuals, [36, 37] ignored the health effect of LTCI policy, and the improvement of health quality is one of the most direct objectives of LTCI. Therefore, we consider the first batch of national LTCI pilot areas as the main study body, based on the three phases tracking survey data of China Health and Retirement Longitudinal Study (CHARLS). The CHARLS deeply analyzes the policy effect of the LTCI pilot to provide reliable support for the policy adjustment and optimization of the LTCI pilot. The main marginal contributions of this study focus on the following points: First, taking 15 national LTCI pilot cities as the main body, we investigate the impact of the policy pilot on the income fairness of group system and thus expands the research viewpoints of existing studies. Second, based on the policy pilot and taking the economic and health effects as the core content, we comprehensively analyze the institutional effect of the LTCI policy pilot. Moreover, the research method uses both the DID and instrumental variable (IV) method to test the institutional effect of policy pilot. Additionally, it investigates the economic adjustment effect of family income level, residence registration, and basic medical insurance on the LTCI policy pilot.

## Methods

### Benchmark regression model

**Fig. 1:**
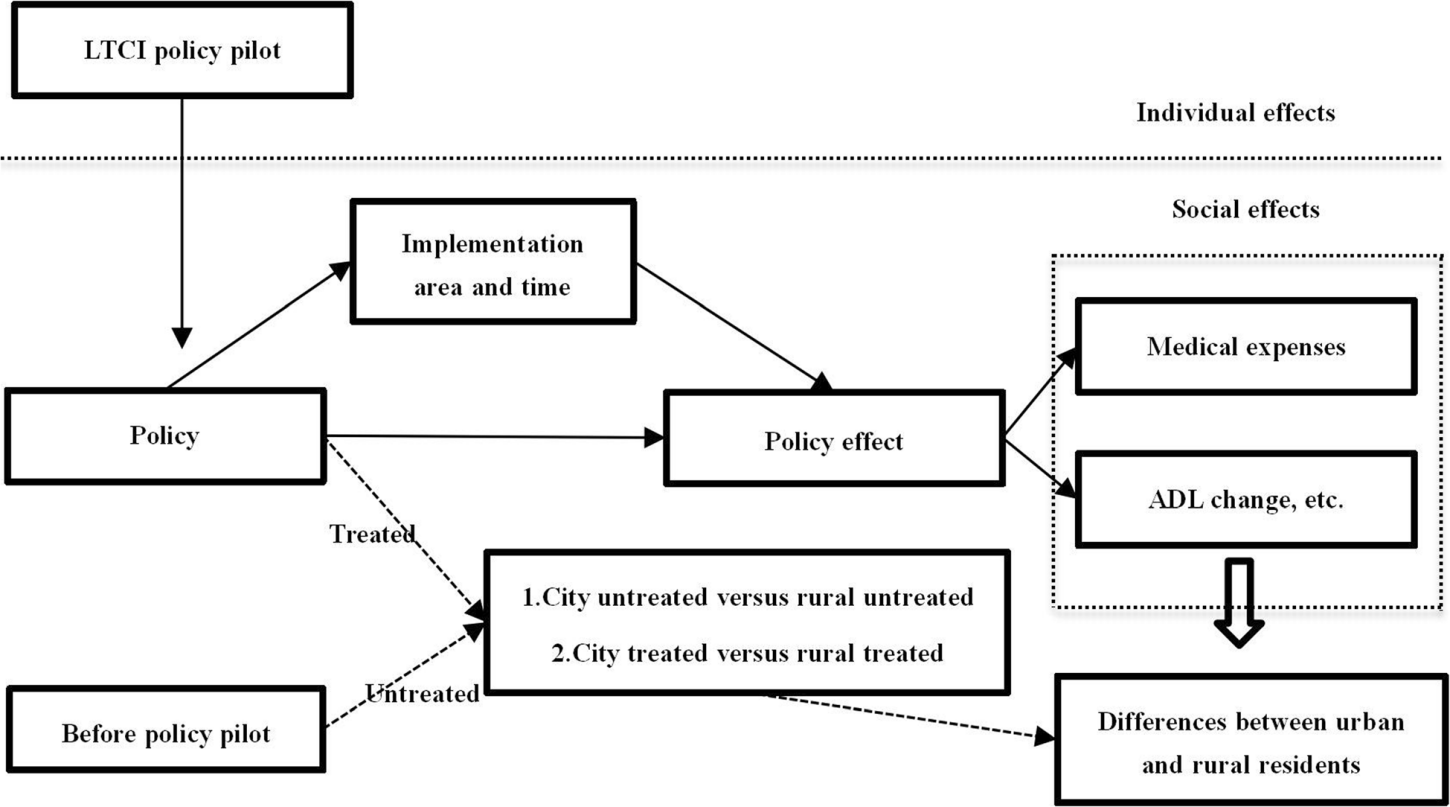
Effect diagram of LTCI policy pilot of China (Differences between urban and rural residents)

In this study, the differences-in-differences (DID) method is used to identify the impact of the pilot policy of LTCI on the equity of medical expenses and health level of the elderly. Moreover, Fig. 1 shows the theoretical derivation of LTCI pilot. The benchmark model is set as follows:

1 $$ {Y}_{ij t}=\alpha +{\alpha}_1{Shidan}_{ij}\times {policy}_{it}+\beta {X}_{ij t}+{\tau}_t+{\omega}_i+{\varepsilon}_{ij t} $$

Where *i* represents individual; *j* indicates group; *t* represents time. In Eq. (1), *Y*_*ijt*_ represents the explained variable, that is, the outcome variable of the individual in the grouping, which includes both the medical expenses (distribution) variable and the health equity variable. To ensure the reliability of the test results of economic effects, we also added the interaction effect between DID and individual residence registration, income class and basic medical insurance. The economic effect model essentially satisfies the consumption function theory, wherein income is an important restrictive factor of consumption. However, China’s household register benefits and the basic medical insurance categories are different because of the fragmentation of the system. In China, especially, basic medical insurance and household register are two important regulating variables.

In Eq. (1), *Shidan*_*ij*_ × *policy*_*it*_ represents the interaction of national LTCI pilot city and treatment group before and after the policy, and coefficient *α*_1_ is the focus of this study. According to data availability, this study sorted out the data of some pilot cities of LTCI. Because the CHARLS survey area did not cover Changchun, Nantong, and Shihezi, while the other 12 cities were covered, we selected 12 pilot cities covered in the sample for analysis. In 2016, the national LTCI pilot was officially implemented. Therefore, we defined the data of pilot areas in CHARLS in 2018 as post-processing, and before 2018 as pre-processing. We then determined it as the corresponding value after processing according to policy implementation time of the pilot areas. Basically, *policy*_*it*_ = 0 if before policy pilot, and *policy*_*it*_ = 1 if after the policy pilot. Among pilot cities, Qingdao is special. The LTCI pilot began in 2011; however, the system only covered rural residents in 2015. Therefore, overall planning type is defined according to time node. Combined with the collection and payment methods of LTCI in pilot cities, we determine the participation of individual LTCI in different regions according to the basic medical insurance types they participate in or enjoy. Among them, urban employees are identified according to the participation of employee medical insurance. Urban and rural residents are identified according to the basic medical insurance for urban residents, the new rural cooperative medical system, and the medical insurance for urban and rural residents. *X*_*ijt*_ represents individual covariates, which includes demographic characteristic variables such as individual gender, age, and marital status (Eq. 1). Socioeconomic status variables such as education and family income level and self-rated health, ADL disability level, number of serious diseases, and other health-level variables. *τ*_*t*_ and *ω*_*i*_ in the benchmark model represent time and individual fixed effects, respectively, and *ε*_*ijt*_ represents random disturbance term.

### Inequality measurement index setting

The index of inequality is selected as the key index by using the Concentration index (CI) and Theil index (TI). Hence, we investigate the medical expenses and health equity of urban residents with different household register and income levels under the LTCI policy.

#### Concentration index (CI)

The concentration index is consistent with the Gini coefficient, which is the generalization of Lorentz curve. Essentially, it reflects the proportion of people at different income levels and the difference in the proportion of resources, and its value range is - 1 ~ 1. On the trend chart, the concentration index is also a curve from left to right. Generally, it reflects the situation that different proportions of people that occupy resources. When it is absolutely equal, it is a 45 ° line. The calculation formula of concentration index that reflecting the area below the slash is:

2 $$ S=\left(1/2\right)\sum \limits_{j=1}^j\left({B}_{j-1}+{B}_j\right)\left({A}_{j-1}+{A}_j\right) $$

In Eq. (2), *A*_*j*_ is the cumulative percentage of population in group *j* and *B*_*j*_ is the cumulative percentage of statistical indicators in group *j*, such as the cumulative percentage of medical expenses and health level involved in this study. On index meaning, if the concentration index is positive, it indicates that medical reimbursement and high health level are beneficial to high-income groups, that is, they have reverse (regressive) distribution effect, and the larger its coefficient, it indicates that the institutional effect is more “Pro rich”. If the concentration index is negative, it indicates that medical reimbursement and high health level are beneficial to low-income groups, that is, they have a positive (progressive) distribution effect, and the smaller the negative value, the more “Pro-poor” the institutional effect is.

#### Theil index (TI)

The Theil index measures income inequality between individuals or regions, which is also known as Theil’s entropy measure. Considering computational advantage, Theil index can not only reflect group inequality but also measure the contribution of intra-group gap and inter-group gap to the total gap. Different from the high sensitivity of Gini coefficient to measure inequality among middle-income people, Theil entropy t index, l and V index, are more sensitive to the changes of upper income and lower income, respectively, to engender complementarity. Here, they are, set as follows, respectively:

3 $$ T=\frac{1}{n}\sum \limits_{i=1}^n\frac{y_1}{\overline{y}}\log \left(\frac{y_1}{\overline{y}}\right)={T}_b+{T}_w=\sum \limits_{k=1}^k{y}_k\log \frac{y_k}{n_k/n}+\sum \limits_{k=1}^k{y}_k\left(\sum \limits_{i\in {g}_k}\frac{y_i}{y_k}\log \frac{y_i/{y}_k}{1/{n}_k}\right) $$

*y*_*i*_ represents the income or health level of individual *i*, and $\overline{y}$ represents the average income or health level of all the individuals (Eq. 3). *T*_*b*_ and *T*_*w*_ are the decomposition of Theil index, representing inequality between groups and within groups, respectively. The rightmost side of Eq. (3) represents the calculation of the decomposition term of Theil index. Generally, the smaller the Theil index, the smaller the degree of inequality, and vice versa.

## Data

The data is selected from the survey data of China Health and Retirement Longitudinal Study (CHARLS) database in 2013, 2015, and 2018. CHARLS data covers samples from 28 provinces, municipalities, and autonomous regions in mainland China. The survey subjects are the population aged 45 and over, which can better reflect the basic characteristics of China’s elderly population. This study selected three follow-up survey data for analysis. Through data screening and selection, 25,063 valid samples with a 3-year period were finally obtained. The core explanatory variable of this study is the pilot of LTCI system, with Table 2 showing the definition and statistics of relevant individual covariates.

**Table 2:**
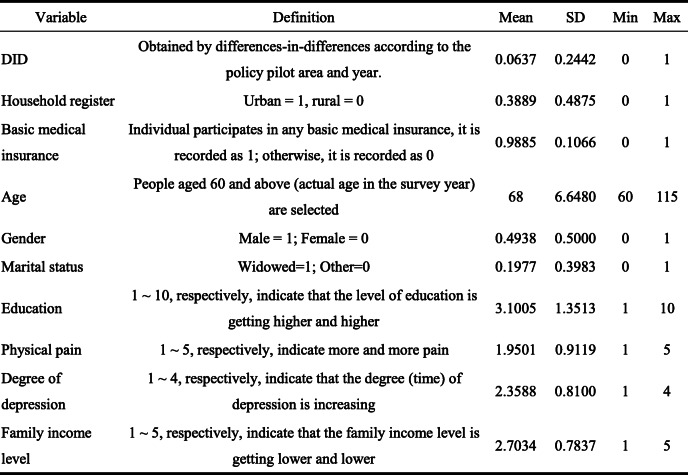
Descriptive statistics of variables (years: 2013, 2015, 2018; place: 28 provinces in China)

With the respect of descriptive statistics, we also focus on the differences between urban and rural areas, focusing on changes in medical consumption and health level of urban and rural residents before and after the pilot of LTCI policy. We aim to explore the impact of LTCI policy pilot on the benefit equity of urban and rural residents. In theory, comparing group differences between urban and rural residents before and after the policy pilot can be divided into four types (Table 3). Among them, the first city untreated versus rural untreated and the second city treated versus rural treated are the focus of this study.

**Table 3:**
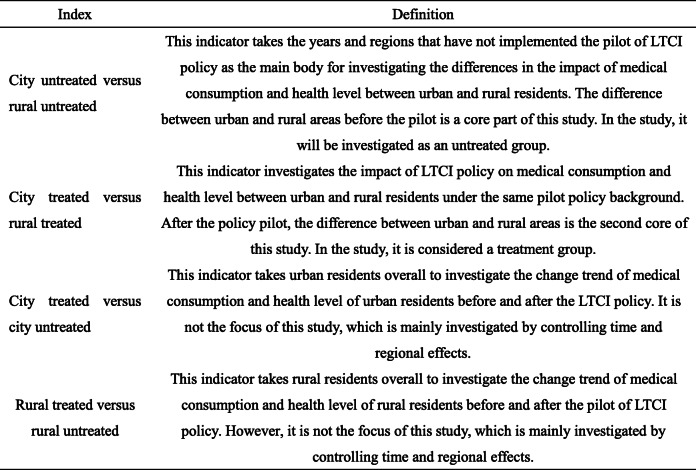
Explanation of corresponding indicators of treatment and untreated groups

We took cities that have implemented the pilot of LTCI policy as the treatment group and determine the sample of residents according to coverage of local policies. Urban and rural residents who in the area that did not implement the pilot policy of LTCI was defined as the untreated group. Because the treatment group includes differences in the policy pilot time of the pilot areas, considering specific treatment, we regard all residents after the pilot time as the treatment group and residents in areas before the pilot policy as the untreated group. Among them, the samples of the treatment and untreated groups are not only the same population but also include residents who have not implemented the LTCI policy before and after policy pilot as part of the untreated group. Therefore, the total number of samples in the processing group is less than that in the untreated group.

Tables 4 and 5, respectively, provide the grouping descriptive statistics of the core explanatory variables of this study, medical expenses, and health level. In the untreated group, the elderly have significant urban-rural differences in monthly outpatient medical reimbursement expenses, annual inpatient reimbursement expenses, and corresponding reimbursement rate (Table 4). Moreover, compared with the rural elderly, the urban elderly have strong advantages in average monthly outpatient reimbursement expenses, annual inpatient reimbursement expenses, and corresponding reimbursement rate. For example, the monthly outpatient reimbursement expenses of the urban elderly is 736.3572 yuan / month higher than that of the rural elderly, and other aspects also show absolute advantages. In the treatment group, significant urban-rural differences in outpatient and inpatient reimbursement rates and annual inpatient reimbursement expenses exist as well; however, no significant difference in the outpatient reimbursement expenses was found. Additionally, compared with the untreated group, differences in outpatient and inpatient reimbursement rates between the urban and rural elderly in the treatment group is narrowing, and their differences in inpatient reimbursement expenses are also narrowing, indicating that the pilot of LTCI has significant impact on the medical expenses of the urban and rural elderly.

**Table 4:**
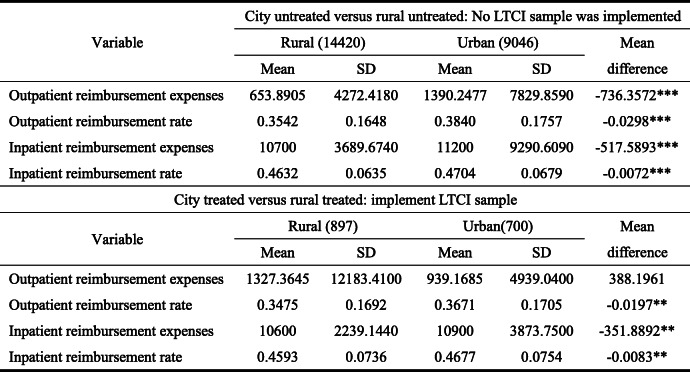
Difference test of medical consumption between urban and rural under treatment and untreated groups (years: 2013, 2015, 2018; place: 28 provinces in China)

On the number of samples in brackets, outpatient expenses are calculated in months, while inpatient expenses are calculated in years

**Table 5:**
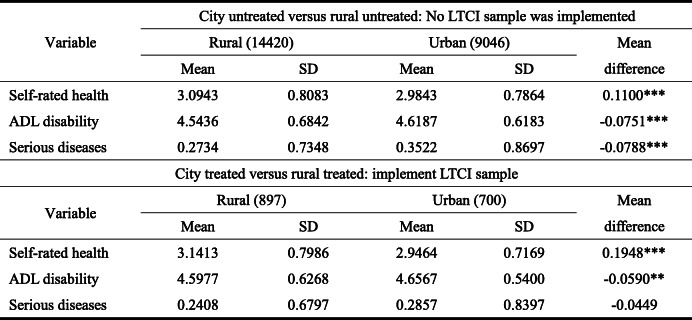
Difference test of health level between urban and rural under treatment group and untreated group (years: 2013, 2015, 2018; place: 28 provinces in China)

On the number of samples in brackets, the value of self-rated health is 1 ~ 5, which means that it decreases from very good to very bad, respectively. The value of ADL disability is 1 ~ 5, indicating severe, partial, moderate, and mild disability, respectively. The number of serious diseases refers to the total number of major diseases diagnosed of the elderly, including malignant tumors, heart disease, stroke, and so on. Larger values reflect worse health quality

Significant differences in health level between the urban and rural elderly in the untreated group exist (Table 5). Among them, the self-rated health level and ADL disability level of the rural elderly are worse than those of the urban elderly; however, the number of serious diseases of the urban elderly is higher than that of the rural elderly. The reason is that the annual physical examination rate of the elderly in rural areas is lower than that in urban areas, resulting in a low overall number. In fact, severe and undiagnosed diseases were found later for rural ones. In the treatment group, compared with the untreated group, significant differences in self-rated health and ADL disability between the urban and rural elderly exist. However, the difference in the number of serious diseases is no longer significant. Additionally, compared with the untreated group, the gap between the urban and rural elderly in self-rated health in the treatment group is widening. The reason is that the self-rated health level of the urban elderly is significantly better, with an average of 2.9464. In contrast, the average self-rated health of the rural elderly after the pilot has increased to 3.1413, widening the difference between urban and rural areas. This is because some pilot areas of LTCI policy mainly cover urban workers or residents, while some areas cover rural residents. However, because of restrictions such as system accessibility, the rural elderly benefit relatively little from LTCI. Considering the ADL disability indicators, ADL disability status of the urban and rural elderly in the treatment group has improved, and its mean values have increased from 4.5436 and 4.6187 to 4.5977 and 4.6567 in the untreated and treatment groups, respectively. Moreover, the overall ADL disability difference between urban and rural residents in the treatment group is also narrowing, from 0.0751 to 0.0590.

## Results

### Economic effect of LTCI policy

#### Economic expenses effect

As the LTCI system was designed to improve the daily care and basic medical care service security of the disabled, it is of great significance to investigate the policy effect of LTCI from the medical expenses perspective. First, the fairness of medical expenses reflects the accessibility and equality of urban and rural individuals or individuals of different ages in enjoying medical services or economic security, which has significant impact on health. Second, considering the LTCI policy pilot, each pilot area has strict evaluation criteria for disabled individuals, such as continuous disability treatment for more than 6 months. Moreover, the pilot LTCI policy aims to inhibit the growing medical expenses, that is, to reduce the payment pressure of the basic medical insurance fund. Therefore, investigating the economic effect of LTCI from the medical expenses perspective provides important practical basis and theoretical support for policy designs. The variables of medical expenses adopted in this study can reflect not only the fairness of medical expenses but the income fairness of different groups in the system to a certain extent.

Table 6 shows the empirical test results. Because subjective factors in the selection of national representative cities in the pilot area of LTCI exist, resulting selective errors in the estimation results. Therefore, to ensure the robustness of the estimation results and avoid the endogenous impact of policy model selection in the pilot areas of LTCI, we select instrumental variables for endogenous treatment. As the basic medical insurance system has national homogeneity in designing the reimbursement system, although regional differences in reimbursement amount and reimbursement rate exist, it generally contains the characteristics of exogenous system. Therefore, we attempt to select the geographical region (location) where the individual is located as the instrumental variable and use the two-stage least square (2sls) method for endogenous processing. Overall, according to the geographical location distribution characteristics of the country’s east, middle, and west, geographical regions are divided into the east, middle, and west, and their corresponding values are 1, 2, and 3, respectively. Table 6 shows the results of the first stage of Model 1. Under Models 2–6, as the overall covariates of the first stage are similar and the results are basically the same, they are not listed here. From the results of the first stage, geographical regions have significant impact on the pilot of LTCI policy, indicating that selection of instrumental variables is effective. Moreover, the F value in the first stage is significantly greater than 10, indicating that weak instrumental variables are not an issue. Additionally, we add 1 to take the logarithm to the variables related to medical expenses to avoid the effects of data non-smoothness and negative after taking logarithm.

**Table 6:**
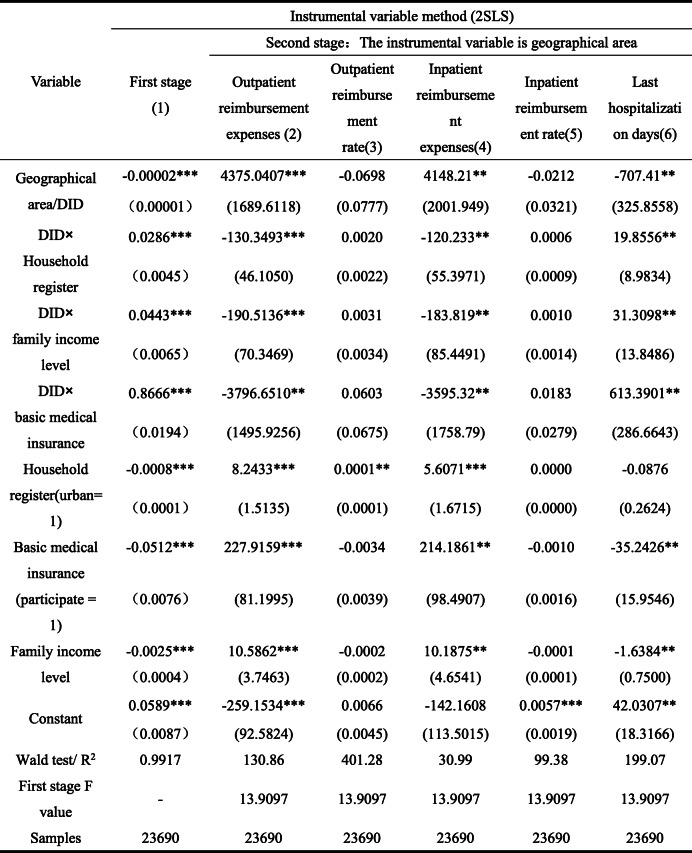
Test results of economic expenses effect (years: 2013, 2015, 2018; place: 28 provinces in China)

Robust standard error in brackets, * * * *P* < 0.01, * * *P* < 0.05, * *P* < 0.1. Here, the explained variables outpatient reimbursement expenses and inpatient reimbursement expenses are logarithmicized. The instrumental variable is the geographical region, which can comprehensively reflect the social and economic environment and other factors. The results of first-stage test confirms its effectiveness. The results of individual health variables ADL, serious diseases, self-rated health, degree of depression, and physical pain as well as age, marital status education, gender, and other variables reflecting individual characteristics are not listed

Considering the role of LTCI policy in Models 2–6, it significantly improves the outpatient and inpatient expenses of the elderly, that is, after the pilot implementation of LTCI policy, the monthly outpatient reimbursement expenses and annual inpatient reimbursement expenses of the elderly have increased by 4375.0407 yuan / month and 4148.21 yuan / year. After implementing the LTCI policy, the hospitalization days of the elderly have been significantly reduced. This is because pilot areas have made strict restrictions on the connection between the enjoyment of LTCI benefits and medical expenses in the LTCI benefits payment policy to avoid the waste of resources caused by repeated payment. LTCI treatment and basic medical insurance treatment cannot be enjoyed simultaneously. Generally, higher inpatient expenses also need relatively high out-of-pocket expenses. Therefore, the length of stay is reduced to a certain extent. Additionally, the disability treatment requirements for more than 6 consecutive months will also impose a certain threshold on this reduction effect; hence, it will not completely reduce medical demand. In Model 4, the positive effect of LTCI policy on inpatient reimbursement confirms this inference.

Considering individual covariates, household register, income level, and basic medical insurance have significant positive effects on the reimbursement expenses of outpatients and inpatients, among which basic medical insurance has the highest positive effect. While length of stay is not significantly affected by household register, the basic medical insurance and income level have significant negative effects, among which the basic medical insurance has the highest reduction effect, reaching 35.2426.

From the second stage interaction item, LTCI policy and household register interaction item significantly reduced monthly outpatient reimbursement expenses and annual inpatient reimbursement expenses and significantly improved the number of hospitalization days for the elderly. At the same time, interaction items between LTCI policy and income level and interactions of LTCI policy and basic medical insurance also show the same characteristics. The results above show that when the pilot scheme of LTCI policy is implemented, household register, income level, and basic medical insurance significantly inhibit the increase of the monthly outpatient reimbursement expenses and the annual reimbursement expenses for elderly patients, it was because LTCI policy significantly increase the number of days of hospitalization for the elderly. Additionally, among regulatory effects, basic medical insurance plays the highest regulatory effect. This indicates that an institutional connection between the LTCI policy and basic medical insurance exists. Moreover, through expense control constraints such as the out-of-pocket expenses of the basic medical insurance, combined with the limitation conditions of LTCI benefits, the policy can effectively alleviate the pressure of continuously increasing medical expenses.

#### Effect of LTCI policy pilot on the inequality of economic distribution

From the concentration index in Table 7, compared with the untreated group, the concentration index of the treatment group is negative. This indicates that a positive distribution effect exists, which allows the elderly with lower income to obtain better outpatient consumption from the LTCI system. Essentially, the policy is pro-poor. In terms of hospitalization days, the concentration index is also negative, indicating that low-income elderly obtain better fairness under the hospitalization limits. Additionally, on outpatient reimbursement rate and inpatient reimbursement expenses, the elderly’s inequality in the treatment group is relatively lower than that in the untreated group; hence, the policy does reduce inequality.

**Table 7:**
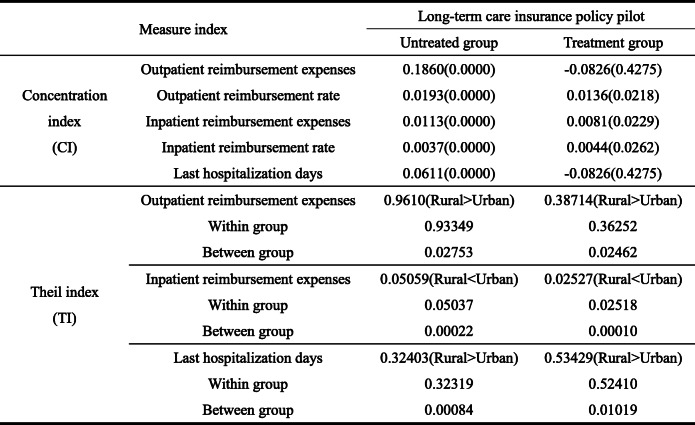
Concentration and Theil indexes of medical expenses under the pilot of LTCI policy (years: 2013, 2015, 2018; place: 28 provinces in China)

*P* value in brackets

Judging from the Theil index of the LTCI pilot policy, the Theil index of outpatient reimbursement expenses in the treatment group is much lower than that in the non-implemented areas, indicating that the LTCI pilot policy promotes the fairness of outpatient treatment for the elderly. But meanwhile, the inequality in rural areas is higher than that urban areas. Basically, whether the LTCI policy is piloted, the inequality of outpatient consumption of the rural elderly is higher than that of the urban elderly, which is consistent. For example, most pilot areas consider the urban elderly as the main treatment expenses object. In contrast, the elderly in rural areas have important objective constraints on treatment enjoyment, such as unequal access to medical service resources. This has also been confirmed in the inequality of hospitalization days. However, considering the inequality of hospitalization days, the pilot policy of LTCI has improved the inequality of hospitalization days for the elderly, which is higher than the inequality index of 0.32403 in non-pilot areas. Moreover, this inequality is mainly attributed to inequality within the group, which is consistent with the previous results, that is, because of the variation of provisions of the pilot policy of LTCI in differenct places. In disability assessment, the applicant should have treatment requirements for more than 6 consecutive months, which will improve the hospitalization needs of the applicant; its inequality will inevitably increase compared with the non-applicants. However, considering inpatient reimbursement expenses, the pilot policy of LTCI further reduces inequality of hospitalization consumption of the rural elderly. The reason is that the disabled elderly pass the disability assessment after continuous hospitalization. Moreover, their income loss caused by inpatient expenses will be compensated through the treatment payment of LTCI. Overall, compared with the non-pilot areas, the pilot policy of LTCI has significantly reduced the inequality of inpatient reimbursement expenses for the elderly in both of urban and rural areas.

### Health effects of LTCI pilot policy

#### Health effects

Consistent with the economic effect, under the health effect model, the possibility of institutional endogeneity also exists. Therefore, the estimation results are from the health effect model that is also treated endogenously (Table 8). In the instrumental variable model, the geographical area of instrumental variables still has significant negative effect on the pilot policy of LTCI in the first stage, indicating that instrumental variables are effective. Additionally, the F value in the first stage is significantly greater than 10, indicating that weak instrumental variables is not problematic. As presented in Table 8, the pilot policy of LTCI still has a significant impact on the health level of the elderly, and the main effect is positive. Here, the LTCI pilot policy significantly reduces the probability of poor self-rated health, improved ADL disability, and reduces the diagnosis rate of serious diseases. Basically, when the LTCI policy is piloted, the self-rated health of the elderly in the pilot area is better, and the level of ADL disability is lower than that in the non-pilot area. Additionally, the number of major diseases has also decreased significantly, which confirms the positive health effect of the pilot policy of LTCI.

**Table 8:**
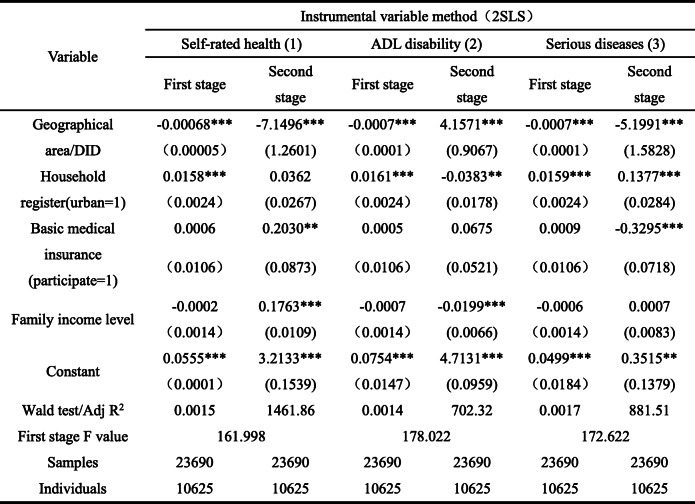
Health effect test results (years: 2013, 2015, 2018; place: 28 provinces in China)

Robust standard error in brackets, * * * *P* < 0.01, * * *P* < 0.05, * *P* < 0.1. The instrumental variable is the geographical region, which can comprehensively reflect the social and economic environment and other factors. The results of one-stage test confirms its effectiveness

Besides, considering individual covariates, household register has a significant and negative effect on ADL disability of elderly people and has a significant and positive effect on the number of serious diseases. This shows that compared with the rural elderly, the ADL disability rate of the urban elderly is higher, and the number of serious diseases is greater. Moreover, basic medical insurance has a positive effect on the self-rated health of the elderly and a negative effect on the number of serious diseases. Essentially, participating in basic medical insurance improves the possibility of poor self-rated health of the elderly. The reason is that compared with the non-participants, objects participating in and enjoying medical insurance have higher expectations for basic medical insurance, and their own health evaluation will be reduced because of their high utilization rate. Thus, it shows a negative effect, which is confirmed by the negative effects on the number of serious diseases. Family income level significantly increased the probability of poor self-rated health and significantly reduced the probability of good ADL, indicating that the higher the income level, the worse the group’s health status. Overall, compared with the health effects of LTCI pilot policy, household register, family income level, and basic medical insurance influence (absolute value of the coefficients) are relatively small, further demonstrating the effectiveness of the LTCI pilot policy.

#### Health concentration index and Theil index of LTCI model

Turning to the concentration index and Theil index of health level (Table 9), in terms of concentration index, the self-rated health of the treatment group is negative, indicating the treatment group improves the health distribution effect of low-income elderly compared with the untreated group. Additionally, considering the number of ADL disabilities and serious diseases, the concentration index of the treatment group is lower than that of the untreated group, further indicating that the pilot policy of LTCI has played a positive distribution role in low-income people to a certain extent. Also, compared with the health concentration index of the untreated group, inequality between different income groups in the treated group was significantly lower.

**Table 9:**
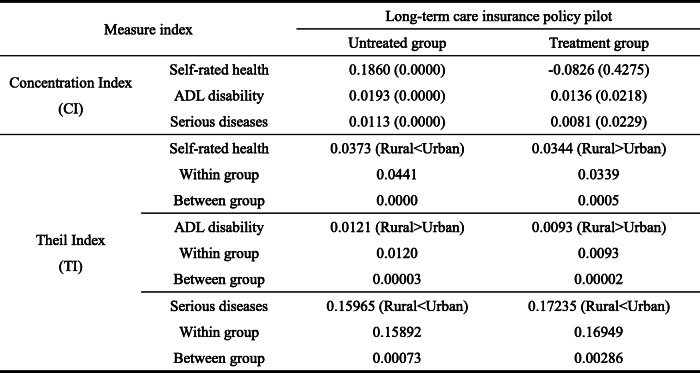
Health concentration and Theil indexes under the pilot of LTCI policy (years: 2013, 2015, 2018; place: 28 provinces in China)

*P* value in brackets

In terms of the Theil index, compared with the untreated group, the inequality index in self-rated health and ADL disability of the elderly in the treatment group is lower; however, the inequality in the number of serious diseases is relatively higher. This shows that the pilot policy of LTCI promotes the fairness of self-rated health and ADL disability of the elderly. Basically, the fairness of health security among groups is guaranteed through the LTCI policy. In practice, the above fairness is mainly through the supply of LTC services in the pilot areas for effectively reducing the deterioration trend of the health status of disabled individuals, which bring about group health fairness. Increase of inequality in the number of serious diseases demonstrates that the pilot of LTCI policy has promoted individuals numbers with LTC services need to apply for disability assessment. Moreover, most regions use the number of serious diseases as an important reference index in disability assessment, which improves the diagnosis rate of the number of serious diseases to a certain extent. Compared with those who did not apply, the disability assessment rate of applicants in each pilot area reached more than 70%. Additionally, the inequality in the number of serious diseases among groups expanded owing to the policy design focusing on protecting the severely disabled. At last, urban-rural differences shows that the inequality of ADL disability of the rural elderly is higher. Inequality in the number of serious diseases among the urban elderly is higher. However, on self-rated health, compared with the characteristics of higher self-rated health inequality of urban employees in the untreated group, rural self-rated health inequality in the treated group is higher. This is mainly because the pilot areas of LTCI policy do not fully include the rural elderly in the system. Although urban and rural planning has been implemented in some areas, LTC service resources have been under-allocated. To some extent, this reduces the health effect of LTCI policy, which confirms the characteristics of higher rural self-rated health inequality.

## Discussion

The pilot of the LTCI policy is an effective measurement for assisting the aging and disability of the population. Moreover, it is also important content for effectively alleviating growing pressure on the basic medical insurance fund. Since the implementation of the national pilot policy in 2016, issues such as the policy effect of the LTCI pilot and the policy adjustments that need to be made in the process of its effective promotion need to be solved. Therefore, beginning from the implementation of regional LTCI pilot, by adopting the CHARLS continuous tracking data in 2013, 2015, and 2018, and based on the perspective of medical expenses and health effect, we empirically investigate the effect of LTCI pilot policies by using differences-in-differences method (DID), Concentration index, and Theil index. Compared with current LTCI policy researches, such as Kim & Lim [14], Choi et al. [18], Yasutake et al. [29], and Ma et al. [13], based on the analysis of LTCI medical cost control utility, this study puts group benefit fairness into evaluation of the implementation effect of LTCI policy for the first time, which is a great breakthrough in research perspective and research content. In respect of research methods, we use the instrumental variable method to reduce the estimation errors caused by sample selection errors, which can effectively improve reliability of research conclusions, which is consistent with the research methods of Boo et al. [16] and Liu [5].

Considering the research results, the current national LTCI pilot has achieved good policy effect; however, many problems in the current policy pilot exist. First of all, compared with the basic medical insurance system, the pilot of LTCI policy needs to be better connected with other social security systems. For example, in the cost control of basic medical insurance, because of strict disability identification conditions, inpatient expenses has been increased to a certain extent. Second, the fairness of LTCI protection objects is also problematic. Most pilot areas take the elderly as the core protection subject, and only protect the severely disabled elderly. However, they have higher coverage in overall funding, that is, there are high inequality of system benefit and unequal “right responsibility ratio” of treatment. Therefore, we need to focus on the focus of the policy pilot. In parallel, starting from the concept of system design, we should not only protect the needs of key groups, but also deal with the excessive “crowding out” of the legitimate rights and interests of the general population, such as the needs of severely and moderately disabled individuals. Moreover, there are deficiencies in the overall planning of the pilot policy of LTCI. On the one hand, most national pilot areas take urban workers as the core guarantee subject, while some areas mainly focus on urban residents and workers. However, they all systematically ignore the LTC services needs of rural disabled individuals, resulting in urban–rural inequality. On the other hand, although some areas have implemented the pilot models of LTCI in both of urban and rural areas, because of the incapacity of LTC services supply, the accessibility of LTC services for disabled individuals in rural areas is insufficient, limiting the effect of policy implementation to a certain extent. Therefore, in the policy pilot, gradually improving the benign development policy of designated LTC services institutions and promoting their regional supply ability of security are necessary.

However, some limitations of this study were noticed. First, although this study investigated the impact of the first round of LTCI pilot policy in China, it did not fully include all the samples of pilot cities, which would affect the reliability of the research conclusions slightly. Second, considering the micro data selection, as the data of this study are selected from the nationwide survey data, although the database includes residents in the pilot policy areas of the LTCI, the pertinence of the research results is relatively insufficient because of the lack of pertinence of the questionnaire survey. Also, considering that the health effect of the pilot of LTCI policy is mainly the improvement of residents’ ADL disability status, because of the lack of direct sample data, it has a certain impact on the research results.

Additionally, from the perspective of the objectives of the pilot policy of LTCI, on the one hand, it is to reduce the rising trend of public medical expenses, on the other hand, it is to promote the standardized development of LTC services security for the elderly or disabled individuals. Therefore, LTCI should show many aspects in the effect of the policy, such as the development of relevant care service institutions, changes in the number and quality of personnel care service, convenience and accessibility of handling services, and the integration and standardization of the financing channels of the system itself. However, because of the limitations of data acquisition, this study did not obtain the detailed development data of designated service institutions and handling institutions. Hence, limitations in the evaluation of the policy effect of this study exist. This will also be the focus of the next research or an important research direction that can be explored.

## Conclusions

As a consequence, first, compared with the untreated group, the difference between the outpatient and inpatient reimbursement rates of the urban and rural elderly in the treatment group is narrowing. While compared with the untreated group, the self-rated health gap between the urban and rural elderly in the treatment group is widening.

Second, concerning the role of LTCI policy in economic impacts, the pilot policy of LTCI has significantly and positively improved the outpatient and inpatient expenses of the elderly and reduced the length of their hospitalization. In terms of health effects, the pilot of LTCI policy has significantly improved the overall health level of the elderly. Among them, the positive impact on the self-rated health of the elderly is the highest, and the impact on the number of serious diseases and ADL disability is second and third, respectively.

Third, our measurement results of inequality show that, from the aspect of economic impact, the concentration index of outpatient reimbursement and hospitalization days of the elderly in the treatment group is negative. Hence, the policy is beneficial to the low-income population. Moreover, the concentration index of inpatient consumption and reimbursement is also relatively low. The Theil index shows that the elderly in the treatment group have relatively lower level of inequality in outpatient and inpatient reimbursement expenses. However, they show higher characteristics in inpatient inequality. Additionally, urban–rural inequality has not fundamentally changed because of the pilot LTCI policy. From the aspect of health effect, concentration index of self-rated health of the elderly in the treatment group is negative. Moreover, concentration index of ADL disability and numbers of serious diseases of the elderly in the treatment group has also decreased significantly. On the Theil index, compared with the untreated group, the inequality of self-rated health and ADL disability of the elderly in the treated group was lower. However, the inequality of the number of serious diseases was higher, and serious diseases here are mainly the number of diseases, not the incidence rate. The reason is found in the adverse selection effects caused by the policy pilot. Conversely, LTCI will improve the survival rate of beneficiaries, which will also increase the prevalence rate. Moreover, on urban-rural differences, the pilot policy has improved the self-rated health equality between the rural and urban elderly. The self-rated health inequality of the rural elderly is more serious, and inequality of ADL disability is also increasing. However, compared with the untreated group, rural elderly in the treatment group showed lower inequality in the number of serious diseases.

## Data Availability

Not applicable.

## Abbreviations

LTC: Long-Term Care
LTCI: Long-Term Care Insurance
CI: Concentration index
TI: Theil index
ADL: Activities of Daily Living
BADL: Basic Activities of Daily Living
IADL: Instrumental Activities of Daily Living
